# Sustainable CO_2_ Capture Using Porous CuBDC Monoliths via Pickering Foam Templating Reinforced with Bacterial Cellulose

**DOI:** 10.1021/acs.langmuir.5c06452

**Published:** 2026-02-09

**Authors:** Zhenghao Shi, Man Hin Kwok, Yifeng Sheng, To Ngai

**Affiliations:** † Department of Chemistry, 26451 The Chinese University of Hong Kong, Shatin, N. T., Hong Kong 999077, China; ‡ School of Chemistry, Chemical Engineering and Life Science, 12565 Wuhan University of Technology, Wuhan 430070, China

## Abstract

Metal–organic frameworks (MOFs) offer high porosity and tunable chemistry, while practical applications are often hindered by their poor processability, low packing density, and inadequate mechanical stability when used in powder form. Shaping MOFs into monoliths could dramatically solve these limitations. However, traditional methods such as sol–gel synthesis, freeze-drying, casting, or templating often involve multiple steps or organic solvents, leading to structural collapse and loss of intrinsic porosity. To overcome the aforementioned challenges, herein we report a green, one-step strategy for fabricating hierarchically porous MOF monoliths via Pickering foam templating. By using hexanoic acid (HA) to in situ modulate the surface of CuO nanoparticles (NPs), ultrastable aqueous foams could be directly prepared while subsequently serving as templates for in situ MOF conversion and growth at the air–water interface. In this work, two typical MOF monoliths based on CuBDC and HKUST-1 were synthesized by this method without the use of surfactants, polymers, or harmful solvents. Besides, backbone materials, such as bacterial cellulose (BC), could subsequently be introduced as a reinforcing scaffold to improve mechanical integrity. Structural analyses revealed that the resulting CuBDC monoliths exhibited well-defined hollow spherical shells templated from the foam bubbles, and the incorporation of BC significantly enhanced compressive strength while preserving hierarchical porosity, although the excessive BC slightly caused pore collapse and surface area reduction. The monoliths showed great potential for CO_2_ adsorption achieving the highest uptake of 2.42 × 10^–1^ mmol g^–1^ at 298 K. This study presents the first demonstration of using Pickering wet foam as a direct template for MOF monoliths, offering a sustainable and tunable approach for scalable fabrication of porous materials suitable for gas storage, separation, and adsorption applications.

## Graphical abstract

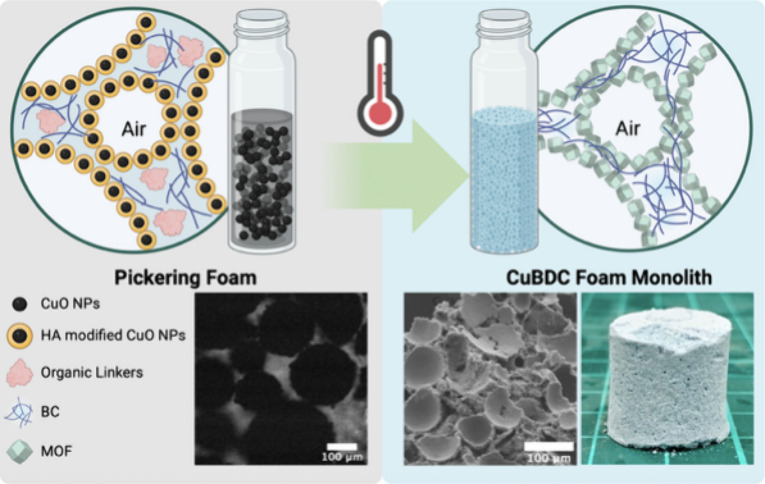

## Introduction

Metal–organic frameworks (MOFs) are a class of porous crystalline hybrid materials composed of metal ions or clusters interconnected by organic linkers through coordination bonds, forming one-, two-, or three-dimensional structures with permanent porosity.^1,2^ Since the late 20th century, following Yaghi’s pioneering report on MOFs, explosive growth has occurred in synthesizing novel MOFs, exploring their underlying mechanisms, and expanding their application scope.^3–6^ In contrast to typical inorganic microporous materials like zeolites, MOFs possess greater design flexibility, allowing simultaneous optimization of pore structure, surface chemistry, and structural diversity,^3^ which make them promising candidates for a wide range of applications, including gas storage and separation,^7^ energy storage,^8^ water treatment,^9^ environment sensor,^10^ drug delivery,^11^ biomedical imaging,^12^ stimuli-responsive luminescence,^13^ and catalysis.^14^ Nevertheless, a key limitation in most MOF applications is that they are typically developed in the form of crystalline powders.^15,16^ Their inherent rigidity and brittleness lead to poor mechanical stability, making them difficult to handle, shape, pack, or recycle in practical use.^5,17^ In addition, their low packing density limits volumetric efficiency and often leads to abrasion, dust generation, clogging, poor mass transfer, and environmental concerns.^18^ To address these challenges, efforts have focused on integrating MOFs into robust macroscopic forms, such as pellets,^19^ monoliths,^20^ membranes,^21^ and fibers,^18^ while retaining their intrinsic porosity and functionality. Each shaping technique delivers unique characteristics to the resulting structure, influencing its dimensions, form, and functional presentation tailored to specific applications.^22^

Traditionally, densifying and shaping MOF powders into compact monoliths, tablets, or pellets can improve mechanical strength and packing density but often compromises porosity, thereby reducing the accessibility of active sites and limiting performance in functional applications.^23–25^ In contrast, fabricating porous MOF monoliths with hierarchical architectures integrates micro-, meso-, and macropores within a single body, offering improved mass transport and mechanical integrity.^25,26^ To date, a variety of well-established strategies have been developed for fabricating MOF monoliths with hierarchical porosity,^27^ which include conventional approaches such as sol–gel synthesis,^16^ in situ growth on 3D foam material,^28^ 3D printing,^29^ casting,^30^ supercritical fluid processing,^24^ freeze-drying,^31^ phase separation,^32^ and etching.^33^ Representative templating strategies involve emulsion templates,^20^ ice templates,^34^ macroporous polymer templates,^35^ and biological templates.^36^ Different shaping methods rely on various additives that act as scaffolds or binders to enhance structural integrity, which often leads to a reduced MOF content in the final bulk material. Interfacial engineering via emulsion templating enables scalable fabrication of MOF monoliths with high MOF content and tunable pore structures.^20^ Pickering emulsion templating, a particle-stabilized approach, eliminates surfactants and improves structural robustness,^37,38^ though partial entrapment of MOF particles within polymer matrices can reduce accessible porosity and performance.^39,40^ High internal phase emulsions reinforced by cellulose nanofibers (CNF) or bovine serum albumin (BSA) have achieved uniform MOF nanoparticle (NP) distribution and well-defined macropores.^41^ Nevertheless, emulsion templating generally relies on organic solvents and surfactants, and subsequent polymer removal or solvent exchange often collapses the delicate framework and diminishes the intrinsic porosity.

Unlike emulsion templating, conventional solvent-free or water-based routes have been investigated as more sustainable approaches for MOF fabrication, but typically lack hierarchical porous structures.^42–44^ Aqueous foam templating^45,46^ has recently gained increasing attention for producing porous materials with well-developed hierarchical porosity and clean processing conditions. Particle-stabilized foams, also known as Pickering foams, exhibit remarkable long-term stability with lifetime extending over months.^47,48^ This exceptional stability arises from the irreversible adsorption of particles at the air–water interface during wet foam aging, where they achieve critical surface coverage and effectively suppress Ostwald ripening, coalescence, and disproportionation through strong interfacial stabilization.^49,50^ As a result, Pickering foams are highly suitable as templates for porous material fabrication.^51–53^ Currently, MOF monolith preparation via foam templating remains reliant on polymer-based porous substrates.^54,55^ Direct use of Pickering wet foams as templates for MOF monolith fabrication has not yet been reported, since the surface properties of MOF particles are commonly unfavorable for the air/water interface.

As representative and well-studied Cu-based MOFs, CuBDC and HKUST-1 were synthesized via a simple one-step Pickering foam templating (Scheme 1), providing typical yet distinct Cu-MOF systems for validation. Through in situ modification of metal oxide NPs dispersed in water, an ultrastable Pickering foam system was successfully generated with the assistance of hexanoic acid (HA). With the further hydrothermal reaction, the corresponding CuO NPs underwent a mineralization process and generated Cu^2+^ ions progressively and reacted with the linker molecules to form MOF crystals at the air–liquid interface. As the MOF growth continued at the interface, a hierarchical porous structure was preserved by templating the aqueous foam. The final dry MOF monoliths were achieved after further drying at 60 °C under ambient conditions until a constant weight to ensure the removal of residual water and HA. From a green chemistry perspective, this method adheres to sustainability principles by minimizing the use of hazardous reagents and utilizing water and air as primary media. All chemicals employed in this process are essential for MOF formation, ensuring an environmentally benign approach. Besides, BC fibers could be incorporated into the system, enhancing the mechanical strength of the monoliths while not adversely affecting their porosity. The resulting MOF monoliths exhibit promising CO_2_ adsorption potential and offer an advanced strategy for CO_2_ capture beyond postcombustion processes for industrial fuel gas treatment.^56^ Such an approach may offer benefits for industrial implementation and environmental sustainability.^57^ Overall, this work demonstrates, for the first time, the feasibility of directly using Pickering wet foams as templates for the MOF monolith fabrication and highlights the general applicability of this strategy to typical Cu-based MOF systems.

**Scheme 1:**
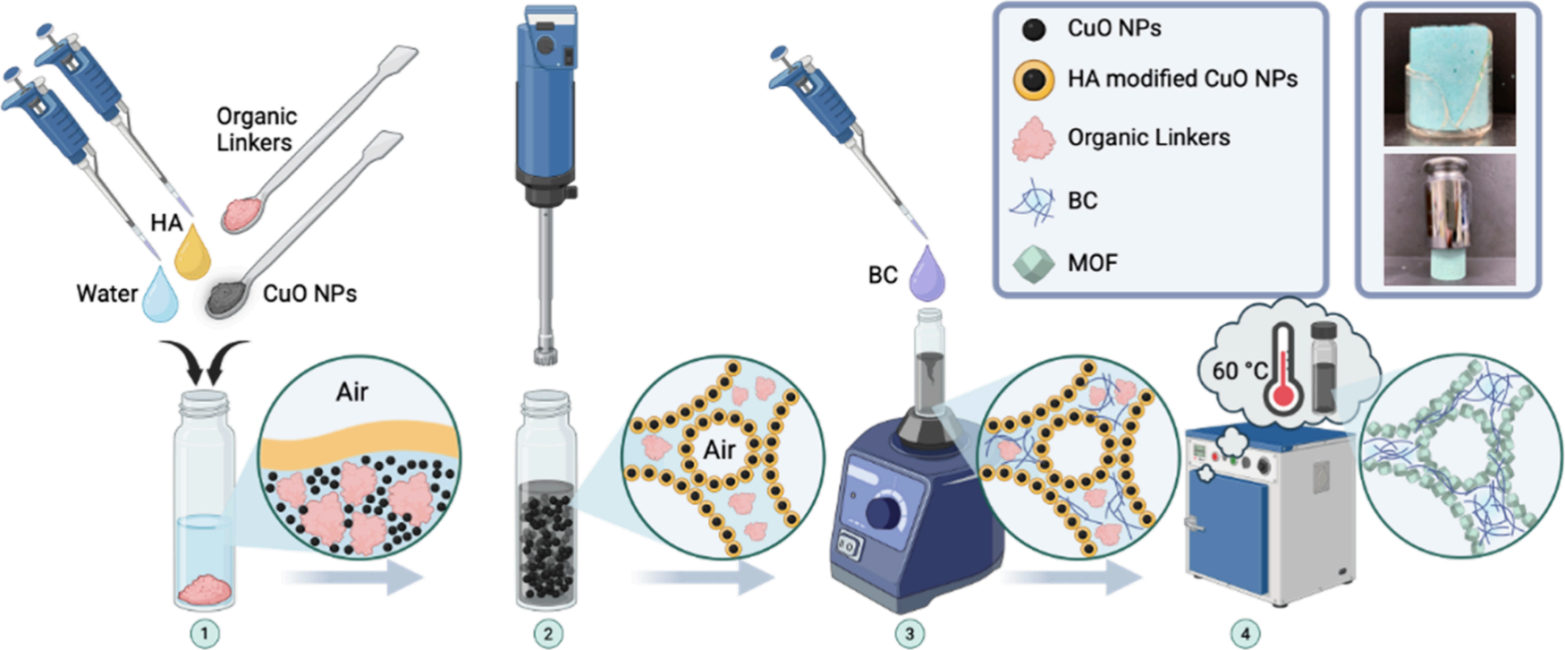
BC-reinforced MOF Monolith Fabrication Process: In the First Step, CuO NPs, Organic Linkers, HA, and Deionized Water Are Subjected to Mechanical Frothing via High-Speed Homogenization to Form a Uniform Pickering Foam^a^

a BC suspension is incorporated into the foam using vortex mixing after foam formation to minimize adverse electrostatic interactions and preserve foam integrity to ensure even dispersion without disrupting the foam structure. The resulting foam mixture is then sealed and incubated at 60 °C to facilitate in situ MOF crystallization at the air–water interface, followed by drying under ambient pressure to yield a porous, reinforced MOF monolith. (Created with BioRender.com.)

## Experiment

### Materials

Commercial copper­(II) oxide NPs with nominal particle sizes of 20, 50, 200, and 1000 nm were obtained from Qingdao Zhongye New Material Technology Co., Ltd. Terephthalic acid (H_2_BDC, >99%) was purchased from TCI Chemical Co., while trimesic acid (H_3_BTC, 98%) was acquired from Acros Organics. HA (*n*-Caproic acid) was sourced from Riedel-de Haën. Bacterial cellulose (BC, aqueous suspension, 0.8 wt %) was supplied by Songhu Shenjian Technology (Dongguan) Co., Ltd. Analytical grade acetone and ethanol were purchased from RCI Labscan Ltd. and Fisher Scientific, respectively. Orthophosphoric acid (85%) was procured from Scharlau Chemicals. Deionized water with a resistivity of 18.2 MΩ cm was obtained from a Smart2 Pure water purification system (Thermo Scientific, Sweden) and used in all experiments. Unless otherwise stated, all reagents were used as received without further purification.

### Synthesis of MOF Monoliths via Pickering Foam Templating

A series of Cu-based MOF monoliths were prepared through a one-pot Pickering foam templating strategy using HA to facilitate the interfacial assembly of CuO NPs at the air–water interface. Two representative systems were explored, including CuBDC and HKUST-1, by employing H_2_BDC and H_3_BTC as organic linkers, respectively. For CuBDC monoliths, 1 g of CuO NPs (50 nm) and 2.0885 g of H_2_BDC (molar ratio CuO:H_2_BDC = 1:1) were weighed into a sample vial, followed by 4.6328 g of deionized water to achieve a total solid content of 40 wt %. Similarly, for HKUST-1, 1 g of CuO and 1.7436 g of H_3_BTC (molar ratio CuO:H_3_BTC = 3:2) were dispersed in 4.1154 g of water. In both systems, HA was added at 4 wt % relative to CuO and preallowed to spread along the surface of the aqueous medium. The mixture was homogenized using an Ultra-Turrax homogenizer (IKA T25 digital, equipped with S25N-18G dispersing tool) for 3 min. Initially, the probe was held at the air–liquid interface and operated at 3000–4000 rpm to facilitate the adsorption of HA onto the CuO surface and promote the formation of CuO–HA complexes at the interface. The probe was then immersed in the bulk solution, and the speed was alternated between 9000 and 10,000 rpm to introduce air and induce foam generation. The probe was vertically moved from the top to the bottom of the vial to ensure a uniform air bubble distribution and proper CuO dispersion across the liquid–gas interface. During this process, CuO NPs, partially hydrophobized by HA, were adsorbed at the air–water interface and stabilized the foam. In contrast, due to their larger powder granule size and poor solubility, the organic linkers predominantly remained in the continuous aqueous phase.

To incorporate BC as a mechanical reinforcement into the preformed CuO–HA–H_2_BDC Pickering foam without compromising foam integrity, vortex mixing was employed using a Vortex-Genie 2 system. Prediluted BC suspensions (0.4 wt %) were added to the as-prepared Pickering foam at three loading levels: 0.5, 1.0, and 1.5 g. These reinforced samples were denoted as CuBDC–BC05, CuBDC–BC10, and CuBDC–BC15, respectively. Vortex mixing was conducted at levels 5–6 (out of 12), corresponding to an estimated rotational speed of 1500–1700 rpm for 15–30 s to ensure adequate dispersion. This moderate-speed vortexing was selected to provide sufficient shear for BC dispersion while minimizing disruption to the delicate air–water interface stabilized by CuO NPs. Notably, the theoretical final CuBDC content in the dried monoliths remained above 99.79 wt %, calculated as 99.93, 99.86, and 99.79%, respectively, based on the mass ratio of CuBDC to the combined mass of CuBDC and dry BC. This ensured that MOF remained the dominant component. All the resulting foams were sealed in glass vials and incubated at 60 °C for 24 h. During this period, the organic linker slowly dissolved and diffused in water, reacting with the gradually mineralizing CuO. MOF crystals nucleated and grew primarily at the air–water interface, forming a shell-like framework that collectively evolved into a three-dimensional, interconnected wet monolith. After crystallization, the samples were dried in a forced-convection oven at 60 °C with open lids to facilitate solvent and HA evaporation. After the drying process was completed until constant weight, the final product was a MOF monolith formed with a hierarchical porous structure via MOF crystal interfacial growth within a Pickering foam template.

### Synthesis of Phosphorylated BC and p-BC-Contained CuBDC Monolith

Since the elemental composition of the original commercial neutral BC comprising only carbon, hydrogen, and oxygen overlaps entirely with that of the organic linkers used in MOF synthesis, it is not feasible to distinguish or spatially resolve cellulose distribution within the MOF matrix via conventional elemental analysis techniques such as energy-dispersive X-ray spectroscopy (EDS). To address this limitation, phosphorylated bacterial cellulose (p-BC) was introduced as a functional alternative, offering a distinct phosphorus signature that can be selectively detected via EDS mapping while maintaining the original hydrophilic character and dispersion properties of BC. Substituting BC with p-BC does not significantly affect the aqueous processing conditions of the CuO–HA–organic linker system. The phosphorylation was carried out by reacting 20 g of a 0.4 wt % BC aqueous suspension with 20 wt % ortho-phosphoric acid (H_3_PO_4_) under continuous stirring at 60 °C for 12 h, enabling covalent attachment of phosphate groups (−OPO_3_H_2_) via esterification. The resulting mixture was diluted 5-fold with deionized water and centrifuged at 9000 rpm for 30 min to remove residual phosphoric acid. This washing process was repeated three times. The purified p-BC was subsequently redispersed in deionized water and recalibrated to 0.4 wt % using mild sonication (5 min, 30% amplitude, pulse mode) to ensure homogeneity and colloidal stability. The phosphorylated BC was then utilized instead of neutral BC in subsequent MOF monolith fabrication, following the procedures described in the Synthesis of MOF Monoliths via Pickering foam Templating section.

### Characterization of CuO NPs and Interfacial Pickering Foam Behavior

The actual particle size distributions and zeta potentials of commercially sourced CuO NPs with nominal sizes of 20, 50, 200, and 1000 nm were evaluated by using dynamic light scattering (DLS) at 25 °C with a Nano ZS90 Zetasizer (Malvern Panalytical, UK). Zeta potential measurements were also conducted across a range of pH values for both CuO NPs and BC to assess their colloidal stability and interfacial interactions under relevant formulation conditions. All pH adjustments for zeta potential measurements and Pickering foam preparations were performed by using a calibrated pH meter (PB20, Sartorius GmbH, Germany). To evaluate the foaming ability and bubble morphology, Pickering foams were prepared using each CuO size in combination with HA, added at 4 wt % relative to CuO. No organic linkers were included at this stage to isolate the contribution of CuO–HA interactions in Pickering foam formation. The resulting foams were qualitatively assessed for foamability and visually examined for bubble size and uniformity with confocal laser scanning microscopy (CLSM) under bright-field mode using a Nikon ECLIPSE C1si microscope equipped with a 20× objective (Nikon Co., Tokyo, Japan). To further investigate the interfacial localization of CuO NPs and organic linkers, fully formulated CuO–HA–organic linker systems were also imaged under the same conditions.

### Characterization of Cu-Based MOF

The crystalline structures of the synthesized MOF monoliths were characterized by X-ray diffraction (XRD), using a Bruker D8 Advance diffractometer (Bruker AXS, Germany) operated at 40 kV and 40 mA. Data were collected over a 2θ range of 5–60° to confirm phase formation and assess the influence of BC incorporation on CuBDC crystallinity. Attenuated total reflectance Fourier transform infrared spectroscopy (ATR-FTIR) was performed using an Alpha FTIR spectrometer (Bruker Optik GmbH, Ettlingen, Germany) in the range of 400–4000 cm^–1^ with a spectral resolution of 4 cm^–1^ to investigate the coordination of organic linkers and detect possible chemical changes introduced by BC addition. The internal porous morphology of the MOF monoliths was visualized by field-emission scanning electron microscopy (FESEM, JEOL JSM-7800F, Japan) operated at 10 kV. To spatially resolve the position of cellulose in the CuBDC framework, EDS (Oxford Instruments, Model 7426) was employed, utilizing phosphorus as a marker element introduced via phosphorylated BC. Mechanical properties were evaluated using a universal testing machine (TOHNICHI, Zhuoyue, Dongguan, China) equipped with a 1 kN load cell to measure the compressive strength of the MOF monoliths before and after BC reinforcement. Thermogravimetric analysis (TGA) was conducted on an HS-TGA-101 system (Hersheng Instrument Technology Co., Shanghai, China) under a nitrogen atmosphere (flow rate: 50 mL min^–1^) with a heating rate of 10 °C min^–1^ to assess the thermal stability of the CuBDC monoliths at various BC loading levels. Specific surface area, pore volume, and pore size distribution were determined by N_2_ adsorption–desorption isotherms at 77 K and CO_2_ adsorption isotherms at 298 K, using an Autosorb iQ MP instrument (Quantachrome/Anton Paar, USA). Before measurement, all samples were degassed under vacuum at 120 °C for 12 h.

## Results and Discussion

### HA-Assisted Metal Oxide-Cellulose Pickering Foam

As a key nanoscale component in the system, the surface characteristics of CuO NPs played an important role in the formation of Pickering foams (Figure 1A). With the dissociation of H_2_BDC, the CuO NP surfaces become positively charged when the pH (pH = 4.2) falls below the isoelectric point, thereby enabling electrostatic adsorption of HA molecules. As a result, the electrostatically absorbance of HA onto CuO NP surfaces with the carboxylate groups, while keeping the hydrophobic alkyl chains extending outward, rendering the particle surface amphiphilic and enhancing interfacial activity. With the synergistic effect of HA and CuO, Pickering foams were formed. In contrast, BC displays a consistently negative zeta potential across the entire tested pH range. Since the fibers aided mechanical reinforcement, it raised the possibility of electrostatic coagulation with the positively charged CuO NPs, which could compromise the structural integrity of the Pickering foam. To circumvent this issue, BC was introduced after the foaming process via brief vortex mixing (15–30 s), thus simultaneously promoting uniform dispersion of BC within the foam matrix and minimizing adverse interactions with CuO NPs.

**Figure 1:**
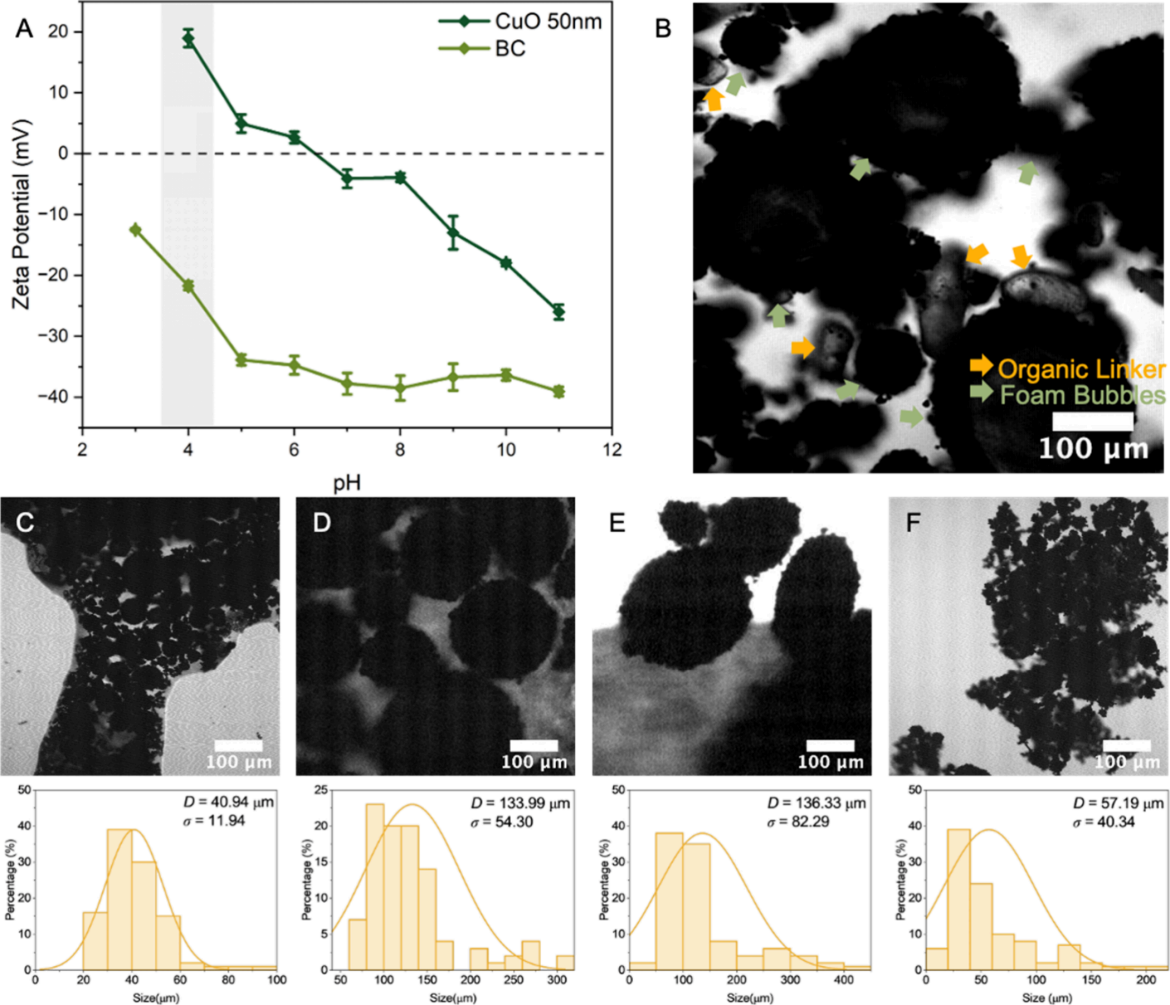
(A) Zeta potential of CuO NPs and BC across pH 3–11. (B) Bright-field image of Pickering foam stabilized by HA-assisted 50 nm CuO and H_2_BDC, showing CuO at the air–water interface. Bright-field images and bubble size distributions (where *D* denotes the mean bubble diameter, and σ represents the standard deviation of the bubble diameter distribution) of foams stabilized by HA-modified CuO at different particle sizes: 20 nm (C), 50 nm (D), 200 nm (E), and 1000 nm (F), without organic linkers.

The microstructure and component distribution of the resulting aqueous foams were visualized via the bright-field mode of CLSM (Figure 1B). HA-modified CuO NPs (50 nm) were observed forming dense, clustered assemblies along the bubble interface, which was consistent with the expected behavior of moderately hydrophobized particles in Pickering foam stabilization. The organic linker H_2_BDC appeared as dispersed transparent microcrystals within the continuous phase due to its poor interfacial activity and large size. When regulating the particle size of CuO NPs (20, 50, 200, and 1000 nm) (Figure 1C–F), it was found that only 50 and 200 nm CuO NPs yielded stable Pickering foams. In contrast, 20 nm CuO NPs demonstrated poor foamability, with significant retention in the continuous phase, due to insufficient interfacial adsorption energy falling below the critical threshold required for stable particle adsorption, compounded by strong Brownian motion and accelerated Ostwald ripening. Conversely, 1000 nm CuO NPs exhibited reduced foamability due to excessive sedimentation driven by gravity, which outpaced their interfacial adsorption. The 50 nm CuO NPs produced more uniform bubbles (σ = 54.30), attributed to their optimal interfacial packing and adsorption kinetics, making them the most suitable candidates for subsequent MOF monolith fabrication.

### Formation of MOF–BC Monoliths and Composition Confirmation

Under mild hydrothermal conditions at 60 °C, CuO NPs modified by HA gradually dissolved to release Cu^2+^, which reacted with H_2_BDC at the air–water interface to form crystalline CuBDC, transforming the foam into an integrated monolith. Verification of CuBDC formation via the Pickering foam templating method was systematically examined by XRD. As shown in Figure 2A, the sample synthesized under mild conditions (60 °C) exhibited well-defined diffraction peaks characteristic of a crystalline CuBDC framework. A strong diffraction peak at 8.47° corresponds to the (001) plane, indicative of the lamellar structure of hydrated CuBDC.^58^ Notably, sharp reflections at 16.2° (011), 20.8° (012), and 23.0° (112) were observed, indicating a high degree of long-range structural order. Among these, the sharpness of the peak at 20.8° particularly signifies high crystallinity.^59,60^ The formation of the MOF was further confirmed by XRD. In contrast to pure CuBDC, the incorporation of BC evidently modified the crystallization behavior of the framework, resulting in composition-dependent changes in diffraction intensity and crystal orientation. By increasing BC content, the intensity of the (011) and (002) reflections at 16.2 and 18.5° increased, respectively. These enhancements may be due to diffraction contributions from BC’s cellulose Iα structure (14.5–16.5°) and newly emerged diffraction planes at the CuBDC–BC interface.^61^ Conversely, the signature CuBDC peaks at 20.8° (012), 23.0° (112), and 26.0° (004) gradually diminished, suggesting that BC incorporation disrupted the interlayer stacking order along the *c*-axis of CuBDC. Additionally, all major diffraction peaks exhibited broader full width at half-maximum (fwhm) with increasing BC content, indicating reduced long-range crystallinity. This decline may arise from spatial confinement of crystal growth within the BC network and hydrogen bonding interactions between BC hydroxyl groups and coordinated water in the CuBDC structure. Interestingly, no distinct peak was observed at 22.5°, a characteristic of crystalline BC, suggesting that BC remained predominantly amorphous in the composite or that overlapping CuBDC peaks masked its signal due to the low BC content and peak broadening.

**Figure 2:**
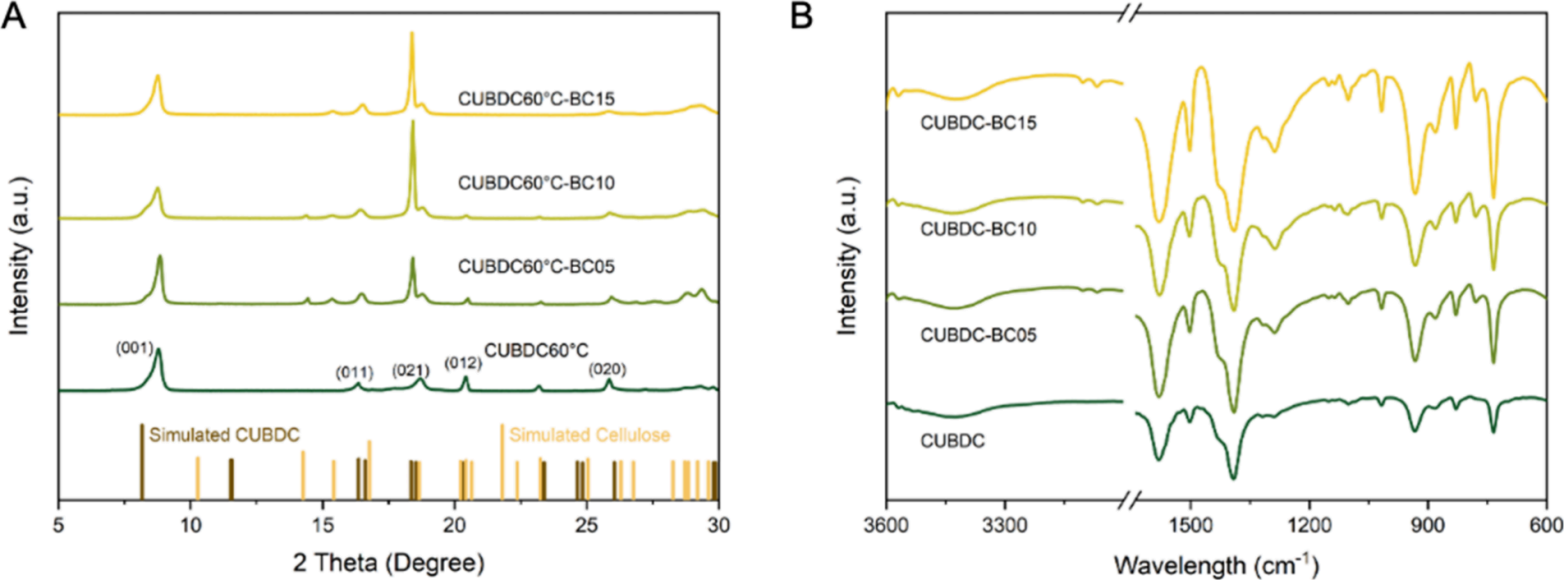
(A) XRD patterns and (B) FTIR spectra of pure CuBDC and CuBDC monoliths reinforced with different amounts of BC. The simulated reference patterns for CuBDC and cellulose were obtained from the Cambridge Crystallographic Data Center (CCDC).

The coordination structure of CuBDC and its composite interaction with BC were also systematically confirmed through ATR-FTIR spectral analysis (Figure 2B). Characteristic peaks observed at 1587 and 1385 cm^–1^ correspond to the asymmetric and symmetric stretching vibrations of the carboxylate group (COO^–^), respectively, indicating that the H_2_BDC was deprotonated and stably coordinated with Cu^2+^ ions.^58,62^ A broad adsorption band in the range of 3500–3200 cm^–1^ is attributed to the O–H stretching vibrations of coordinated or free water, which aligns well with the hydrated structural features of CuBDC and corroborates the interlayer reflection observed at 8.16° in the XRD analysis. The incorporation of BC was further validated by three distinct vibrational signals. The C–O stretching vibration band at 1015 cm^–1^ increased in intensity proportionally with the BC content. The retention of the β-glycosidic linkage peak at 733 cm^–1^ (characteristic of cellulose) confirmed that the crystalline structure of BC remained intact during composite formation, and the broadening of the C–O–C glycosidic stretching band in the 1100–1150 cm^–1^ region suggests interfacial coupling between the amorphous regions of BC and the CuBDC matrix.^63,64^

### Morphology and Porosity Analysis

The morphology of the MOF-based monoliths was further investigated by SEM. Pure CuBDC monolith exhibited a 3D network composed of uniformly distributed hollow spherical shells (Figure 3A). These structures indicated effective templating from Pickering foams, with clearly visible plateau borders between adjacent shells, confirming the templated gas–liquid interface. The integrity of this architecture suggests that the organic linkers successfully diffused into the aqueous phase, enabling CuO dissolution, Cu^2+^ coordination, and localized MOF crystal growth at the air–water interface. However, upon incorporation of BC, the morphological integrity of the CuBDC porous structure was progressively influenced. At a low BC content (CuBDC–BC05, Figure 3B), the spherical shell morphology became less geometrically regular, yet most of the foam-templated cavities and plateau borders were still preserved. Increasing BC content (CuBDC–BC10, Figure 3C) resulted in further structural deformation, and the porous shells began to collapse and merge, resulting in wall-to-wall contact and reduced internal voids. Regarding CuBDC–BC15, (Figure 3D), the structure was dominated by a disordered, loosely packed MOF matrix, with only sparse remnants of the original shell morphology. These morphological transformations can be attributed to BC’s adverse surface changes, which led to the instability of the bubbles. Dispersed BC fibers within the continuous phase of the Pickering foam formed a flexible 3D network-like scaffold. Upon drying, the BC network shrinks and acts like a physical mesh, compressing and locking the MOF-shelled foam bubbles together. While this enhances mechanical strength, BC leads to shell walls merging and plateau regions disappearing when foam bubbles are being compressed. Additionally, the strong electrostatic attraction between the negatively charged BC and positively charged CuO NPs can disrupt HA–CuO interactions, destabilizing the foam and causing a subsequent loss of structural regularity. To further visualize the BC distribution within the CuBDC monoliths, phosphorylated BC (p-BC) was employed in place of neutral BC. SEM–EDS mapping (Figure 3E) revealed colocalization of phosphorus with Cu, C, and O both on the inner and outer shell walls, verifying the successful integration of BC throughout the monolith.

**Figure 3:**
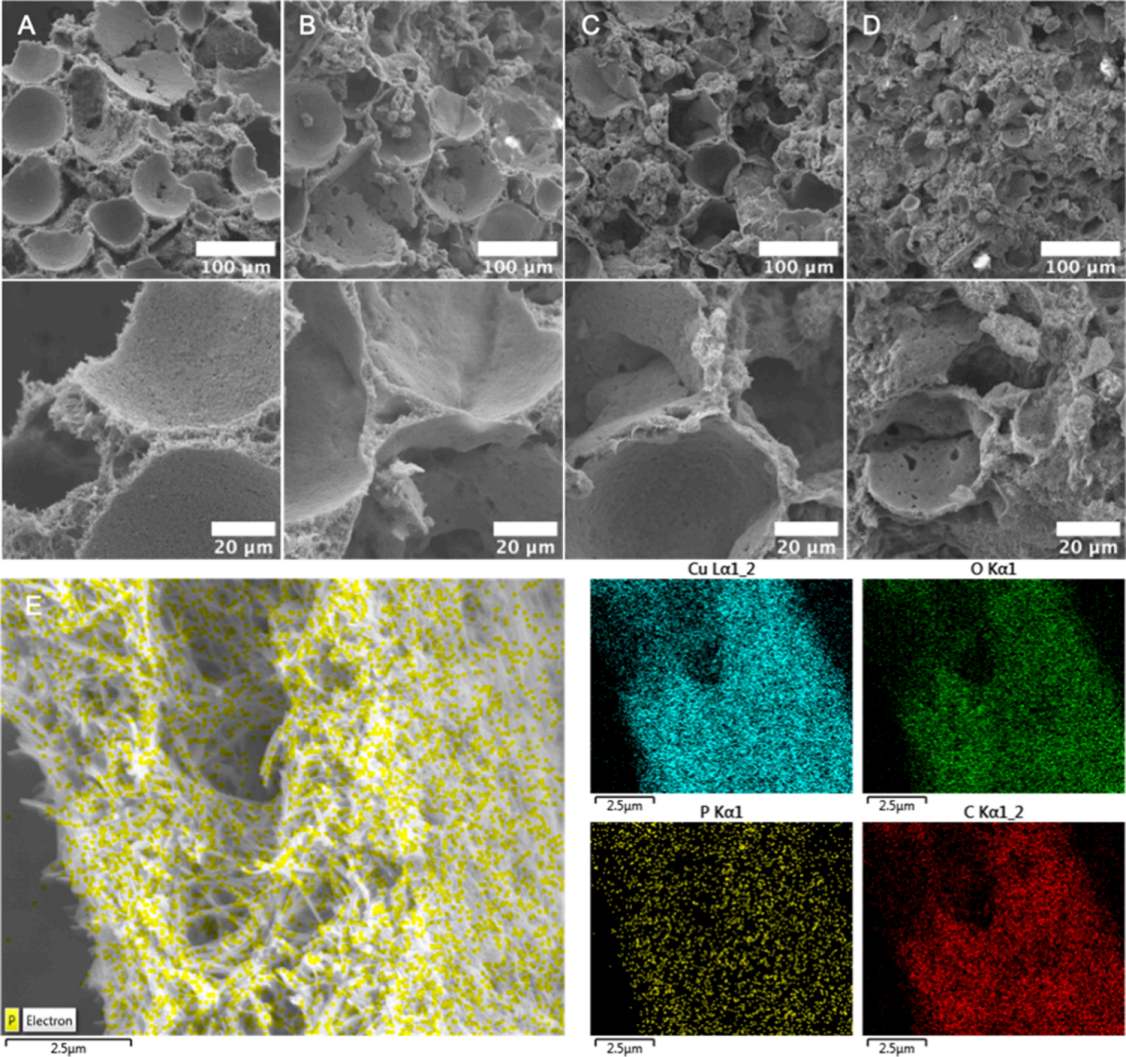
Cross-sectional SEM images of CuBDC and BC-reinforced CuBDC monoliths: (A) pure CuBDC, (B) CuBDC–BC05, (C) CuBDC–BC10, and (D) CuBDC–BC15. (E) Spatial distribution of cellulose visualized by using p-BC as a phosphorus marker.

As a universal strategy, this method can be extended to other systems. To illustrate it, HKUST-1 monoliths were prepared to demonstrate the broader applicability of this Pickering foam templating approach. The observed morphological features from SEM images (Figure 4A–D) revealed parallel trends to those of the CuBDC system. Similarly, the HKUST-1 monoliths displayed foam bubble templated porous structures with thin shells. However, compared to CuBDC, the extent of shell formation was notably reduced upon BC addition, reflecting faster crystallization and weaker interfacial confinement. This difference might be attributed to the higher water solubility and stronger acidity (pH ≈ 3.2) of H_3_BTC, which accelerates CuO dissolution and diminishes the likelihood of MOF nucleation at the air–water interface. As such, this templating method demonstrates greater suitability for MOF systems employing low-solubility organic linkers, such as H_2_BDC, which promote controlled interfacial crystallization and yield structurally well-defined monoliths.

**Figure 4:**
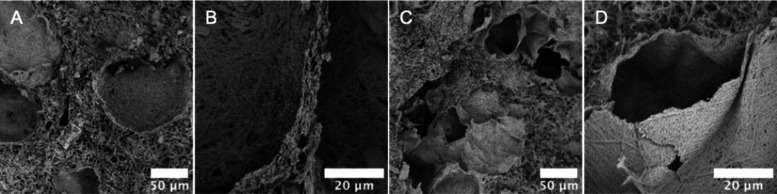
SEM images of HKUST-1 monoliths without BC reinforcement (A, B) and with BC reinforcement (C, D), showing the porous spherical shell structures formed via Pickering foam templating.

### Mechanical Property of MOF–BC Monoliths

It has been proven that monolithic MOF materials with hierarchical porous structures can be successfully prepared using a Pickering foam templating method. This approach helped maintain the overall shape of the material while overcoming common issues seen with MOF powders, such as poor handling and a weak structure. SEM images above showed that the pure CuBDC monolith formed a regular porous structure made of hollow spherical shells. However, because the shells were only loosely connected at the edges (lamellae area of aqueous foam), the overall structure remained weak and lacked mechanical strength. In contrast, when BC was incorporated, a robust network-like scaffold was formed, as the intertwined cellulose fibers along the plateau area of the MOF monolith and interlinked adjacent core–shells provided additional structural support and effectively tightened the porous CuBDC arrangement. TG–DTG spectra of pure CuBDC and CuBDC monoliths reinforced with various contents of BC indicate that BC incorporation contributes to enhanced thermal stability of the CuBDC monoliths (Figure S1 in the Supporting Information), while simultaneously improving mechanical strength without sacrificing overall porosity.

Compression testing (Figure 5A) quantitatively demonstrated the effect of the BC reinforcement. The pure CuBDC monolith collapsed at a relatively low stress of 0.45 MPa. In contrast, the CuBDC–BC05 and CuBDC–BC10 samples exhibited enhanced structural resilience, with failure stresses of 1.04 and 2.40 MPa, respectively, representing mechanical improvements of 131.11 and 433.33%. Importantly, when the BC content was increased further, CuBDC–BC15 exhibited a decline in structural regularity and the formation of loosely aggregated domains, which was indicated in the SEM analysis, leading to a reduced compressive strength of 1.46 MPa. At the same time, the strain-at-break values increased with higher BC content. CuBDC–BC10 and CuBDC–BC15 fractured at 24.27 and 28.51% strain, respectively. Both are higher than that of the pure CuBDC, indicating that the monoliths became less brittle and more ductile as the BC content increased. To further illustrate the reinforcement effect, a weight-loading demonstration was conducted by placing four 500 g counterweights on the monoliths (Figure 5B–E). The results were consistent with the compression tests, in which the CuBDC–BC10 monolith can withstand a total load of 2 kg without visible cracking, whereas the pure CuBDC sample fractured completely under the same conditions, highlighting its poor mechanical integrity. This confirms that BC incorporation substantially enhances the structural robustness and practical applicability of the MOF monoliths.

**Figure 5:**
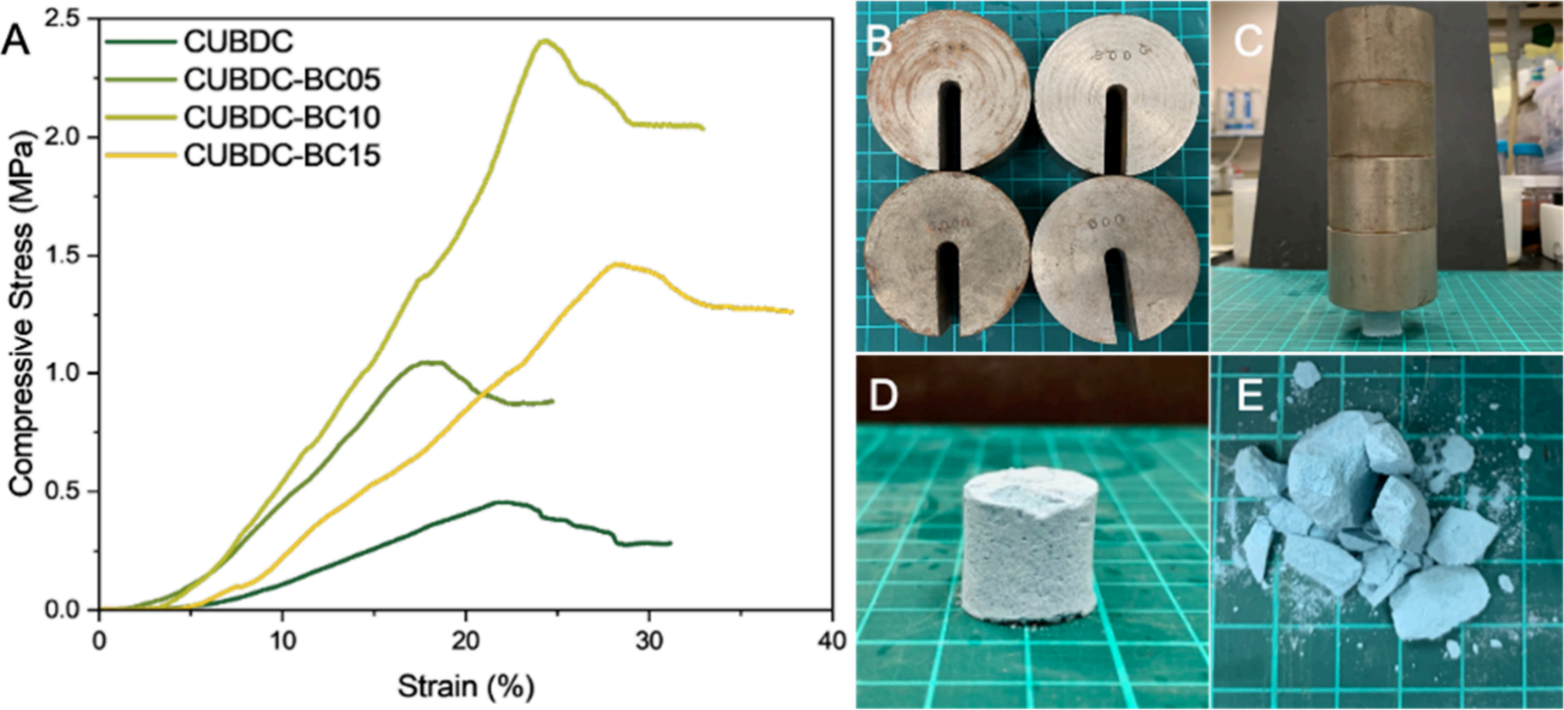
(A) Compression stress–strain curves of pure CuBDC and CuBDC monoliths reinforced with various contents of BC. (B) Four 500 g counterweights for weight-loading demonstration. (C) CuBDC–BC10 monolith supporting four 500 g counterweights without structural failure. (D) CuBDC–BC10 sample after weight loading. (E) Pure CuBDC monolith showing visible cracking after weight loading.

### BET and CO_2_ Adsorption

BET was used to analyze the pore structures of the monoliths, as shown in Figure 6A. The pure CuBDC monolith exhibited a specific surface area of 36.49 m^2^/g, a pore volume of 0.102 cm^3^/g, and a half-pore width of 17.16 Å. Compared with MOF powders, shaped MOF monoliths often display substantially reduced BET surface areas.^23,65^ In foam- or emulsion-templated systems, this reduction is frequently associated with the introduction of binders, polymeric networks, or fibrous reinforcing components (e.g., cellulose) to ensure mechanical integrity, which can partially block micropores and dilute the accessible MOF fraction, leading to BET values in the tens of m^2^ g^–1^ range reported in previous studies.^66,67^ As the BC content increased, CUBDC–BC10 demonstrated an optimal balance between structural reinforcement and textural preservation. Although its BET surface area decreased slightly to 30.92 m^2^/g (retaining around 85% of the original), it displayed the highest pore volume of 0.146 cm^3^/g, a favorable feature for enhancing small guest molecule transport. The half pore width reduced to 9.58 Å, suggesting a denser yet accessible porous framework. In contrast, excessive BC loading in CUBDC–BC15 compromised the porous architecture. The hierarchical shell structure was partially lost, and the excess BC filled interparticle voids, collapsed mesopores, and blocked micropores. This led to a further decrease in surface area to 26.64 m^2^/g and a reduction in pore volume to 0.108 cm^3^/g, ultimately limiting the availability of active sites and mass transport pathways. These findings illustrate the trade-off between reinforcement and porosity and the need to optimize BC content for multifunctional performance. As the material’s intrinsic hierarchical mesoporous network was successfully achieved by Pickering foam templating, the presence of hierarchical pores and high porosity supported its suitability for adsorption and diffusion-based applications.

**Figure 6:**
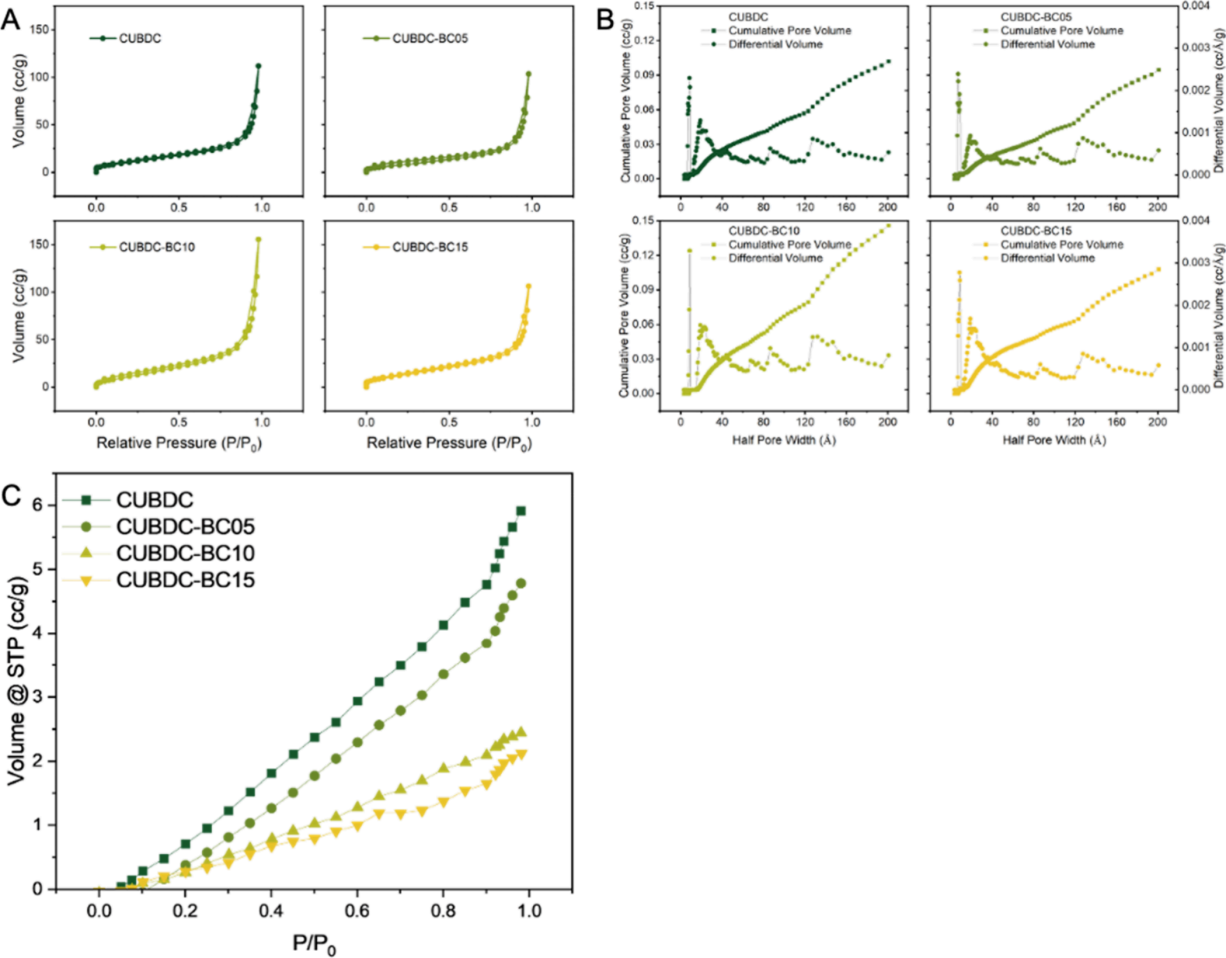
Pure CuBDC and BC-reinforced CuBDC monoliths measured for (A) N_2_ adsorption–desorption isotherms and (B) NL-DFT pore size distributions at 77 K, and (C) CO_2_ adsorption isotherms at 298 K.

As an effective approach for CO_2_ removal and safe storage away from the atmosphere, MOFs have emerged as promising materials due to their tunable pore structures.^68^ To demonstrate the adsorption application, the CO_2_ capture were conducted at 298 K (Figure 6C).^26^ A clear downward trend in the level of CO_2_ uptake was observed with increasing BC content, revealing an inherent trade-off between mechanical reinforcement and porosity preservation. The pure CuBDC monolith exhibited the highest CO_2_ adsorption capacity, reaching 2.42 × 10^–1^ mmol g^–1^ at *P*/*P*_0_ = 0.981, supported by its high surface area (36.49 m^2^/g) and adequate pore volume (0.102 cm^3^/g). In contrast, CuBDC–BC05 and CuBDC–BC10 displayed reduced capacities of 1.95 × 10^–1^, and 9.98 × 10^–2^ mmol g^–1^, respectively, while CuBDC–BC15 showed the lowest uptake at 8.67 × 10^–2^. This decline in performance correlates with reductions in surface area and disruptions in the pore structure. Although CuBDC–BC10 featured the highest pore volume (0.146 cm^3^/g), it exhibited further reduction in CO_2_ uptake. Its adsorption capacity was reduced due to narrowed pores and partial obstruction, resulting from the excessive incorporation of BC. CuBDC–BC15 demonstrated the lowest surface area (26.64 m^2^/g) and notable pore volume shrinkage, indicative of pore collapse and potential cellulose aggregation, which collectively hindered gas diffusion and limited accessible adsorption sites.

## Conclusions

A green and scalable Pickering foam templating strategy was developed for fabricating hierarchical porous MOF monoliths reinforced with BC, successfully addressing the critical challenge of integrating mechanical robustness with preserved porosity in MOF macroscopic assemblies. HA enabled the interfacial activity of CuO nanoparticles at the air–water interface, facilitating MOF formation directly within the foam matrix without the need for organic solvents or complicated processing steps. The resulting monoliths featured spherical shell architectures templated from aqueous Pickering foams, while the incorporation of BC provided mechanical reinforcement, as its 3D fibrous network supported the CuBDC monolith and shrinkage during drying tightened the shell structure. This synergistic effect significantly enhanced mechanical integrity, with the CuBDC–BC10 sample exhibiting a 433% increase in compressive strength (from 0.45 to 2.40 MPa) compared to the unreinforced CuBDC. Our results also showed that the MOF–BC would maintain their good porosity and high connectivity, which could be further used as CO_2_ capture materials. This work marks the first successful use of Pickering wet foam as a direct template for the MOF monolith synthesis. The solvent-free, process-friendly approach enables interfacial MOF growth with tunable porosity and minimal environmental impact. Its simplicity, compatibility with aqueous processing, and structural control make it a compelling platform for advancing porous MOF-based materials in gas storage, separation, and adsorption-related applications.

## Supplementary Material



## Notes

TG-DTG spectra of pure CuBDC and CuBDC monoliths reinforced with various contents of BC (PDF)
