# Supplementary material for: Sustainable CO2 Capture Using Porous CuBDC Monoliths via Pickering Foam Templating Reinforced with Bacterial Cellulose
Source: Langmuir. 2026 Feb 9;42(7):5812–22. doi: 10.1021/acs.langmuir.5c06452 (PMC12951622; doi:10.1021/acs.langmuir.5c06452)
Supplement: Supplementary file 1 [file la5c06452_si_001.pdf]

Supporting Information

Sustainable CO_2 Capture Using Porous CuBDC Monoliths via Pickering Foam Templating Reinforced with Bacterial Cellulose

Zhenghao Shi[a], *Man Hin Kwok*[a], *Yifeng Sheng*[b,*], *To Ngai*[a,*]

[a]Department of Chemistry, The Chinese University of Hong Kong, Shatin, N. T., Hong Kong, 999077, China.

[b]School of Chemistry, Chemical Engineering and Life Science, Wuhan University of Technology, Wuhan, 430070, China.

* Corresponding authors: tongai@cuhk.edu.hk & shengyifeng@whut.edu.cn

Keywords: Pickering Foam, Metal-Organic Framework, Porous Material, CO_2 Adsorption

Contents

Supporting Information 2

Figure S1. TG-DTG of pure CuBDC and BC reinforced CuBDC monoliths. 2

Supporting Information

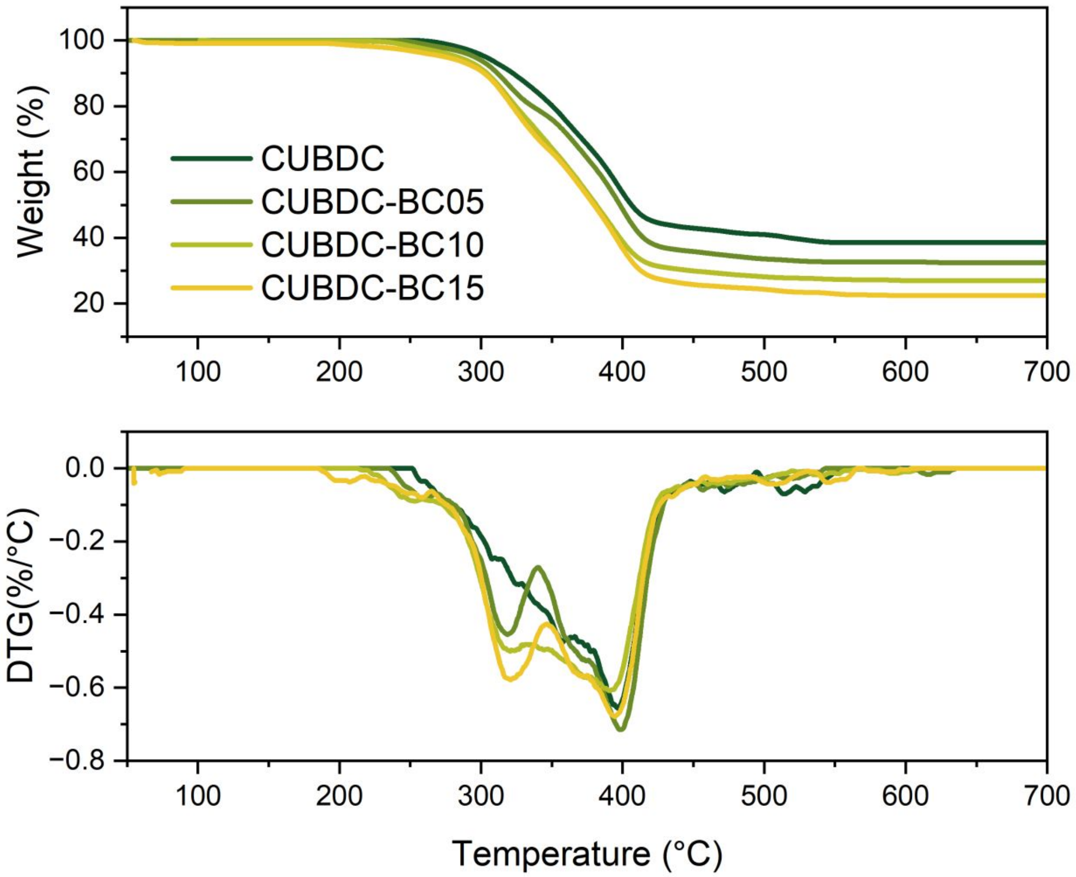

Figure S1. thermogravimetric analysis (TG-DTG) of pure CuBDC and CuBDC monoliths reinforced with various contents of BC.

The thermal stability of CuBDC monoliths was evaluated by TG-DTG analysis under a nitrogen atmosphere from room temperature to 700 °C (**Figure S1**). The pure CuBDC monolith shows a main weight loss beginning at 262 °C, corresponding to decomposition of the organic linker. BC-containing samples exhibit an additional low-temperature mass loss (60-80 °C) due to the hygroscopic nature of BC, followed by cellulose-related degradation between 300-370 °C. Notably, the main DTG peaks of the BC-containing monoliths shift toward higher temperatures (around 400 °C), indicating enhanced thermal stability upon BC incorporation.
